# Anastomotic fart: a ubiquitous general surgical term not described in literature demonstrated in a case report

**DOI:** 10.1093/jscr/rjag096

**Published:** 2026-03-07

**Authors:** Phoebe Bardoul, Stephen French, Tom Burton

**Affiliations:** Department of General Surgery, Nelson Hospital, 98 Waimea Road, Nelson 7010, New Zealand; Department of General Surgery, Nelson Hospital, 98 Waimea Road, Nelson 7010, New Zealand; Department of General Surgery, Nelson Hospital, 98 Waimea Road, Nelson 7010, New Zealand

**Keywords:** anastomotic fart, anastomosis, bowel resection, pneumoperitoneum

## Abstract

Post-operative pneumoperitoneum following abdominal surgery usually resolves within one week but may persist for 10 to 24 days (Gayer G, Hertz M, Zissin R. Postoperative pneumoperitoneum: prevalence, duration, and possible significance. *Semin Ultrasound CT MR*. 2004;25:286–9). However, a new or enlarging volume of pneumoperitoneum following bowel resection is almost inevitably a sign of catastrophe (Gayer G, Hertz M, Zissin R. Postoperative pneumoperitoneum: prevalence, duration, and possible significance. *Semin Ultrasound CT MR*. 2004 Jun;25(3):286–9, Chiarello MM, Fransvea P, Cariati M, Adams NJ, Bianchi V, Brisinda G. Anastomotic leakage in colorectal cancer surgery. *Surg Oncol*. 2022;40:101708). Operative management is usually required in these cases (Chiarello MM, Fransvea P, Cariati M, Adams NJ, Bianchi V, Brisinda G. Anastomotic leakage in colorectal cancer surgery. *Surg Oncol*. 2022;40:101708). ‘Anastomotic fart’ is a commonly used phrase in General Surgical departments, often used to denote an anastomotic leak of gas only and minimal to no faecal contamination of the peritoneal cavity. However, this term is devoid in any published literature. In this case we demonstrate an ‘anastomotic fart’ managed conservatively by describing a 90 year old man who developed a new massive pneumoperitoneum on Day 10 post-operatively. He was able to avoid further surgical management and was safely discharged back to his own home after 24 days.

## Introduction

‘Anastomotic fart’ is a ubiquitous phrase among General Surgeons to describe leakage of gas with minimal faecal contamination into the peritoneal cavity following a bowel resection with anastomosis.

An anastomotic leak after colorectal surgery is a well-recognized and serious complication, associated with significant morbidity and mortality [1]. Such leaks frequently require interventional management, particularly when a proximal diverting ileostomy was not created at the initial operation [1, 2].

We report the case of a 90 year old male who developed large volume pneumoperitoneum 10 days following an open sigmoid colectomy for recurrent sigmoid volvulus. This was managed conservatively with medical management and the patient was discharged home 24 days post operatively.

## Case report

A 90 year old had experienced recurrent episodes of sigmoid volvulus over the preceding 6 months, each of which were successfully managed with endoscopic detorsion. His medical history was notable for hypertension, low grade idiopathic thrombocytopenia (platelet count 110–120 × 10^9^/L) and a cochlear implant. His surgical history included an appendicectomy and right inguinal hernia repair. He lived independently at home without the need for any support services.

Although initially reluctant to undergo surgical intervention, the patient - following discussion with his family and the surgical team - elected to proceed with a sigmoid resection due to the increasing frequency of volvulus episodes.

The patient proceeded to an open sigmoid resection through a muscle-splitting Llanz incision. About 30 cm of redundant bowel was resected and a hand sewn two layer end-to-end anastomosis was formed with 3–0 polydioxanone suture in a continuous fashion. Intra-operative endoscopic assessment of the anastomosis demonstrated an intact anastomosis with negative leak test.

**Figure 1:**
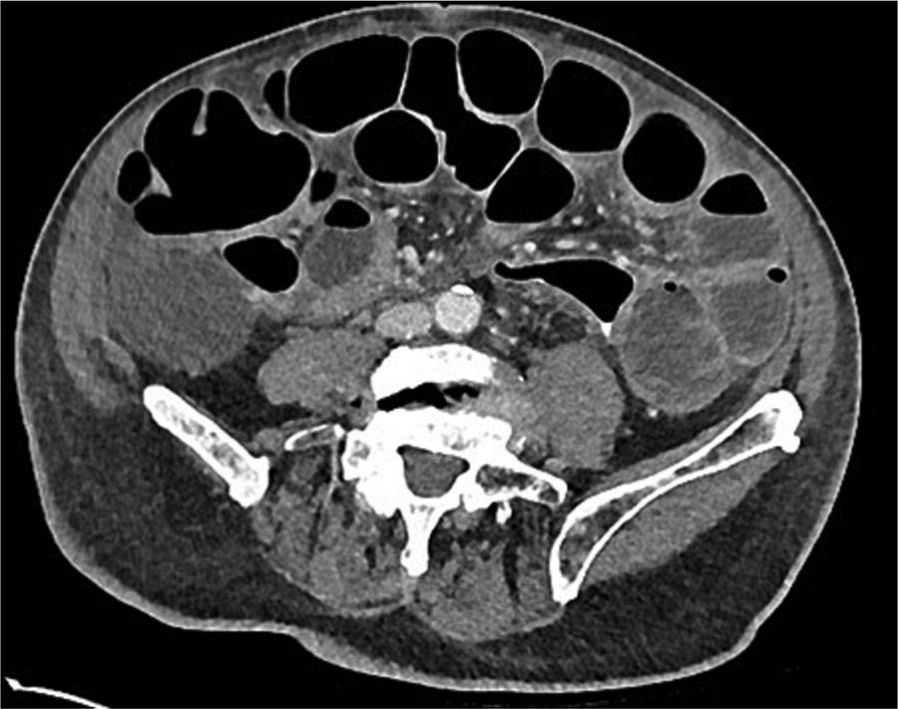
Day 7 CT scan demonstrating features consistent with ileus with no pneumoperitoneum and rectal contrast proximal to anastomosis without contrast extravasation.

Post-operatively, recovery was slow with persistent nausea and vomiting at Day 7. A computed tomography (CT) of the abdomen with rectal contrast was ordered. This demonstrated a 6 cm thickened length of colon with rectal contrast going beyond the area of thickening and no extravisceral contrast (Fig. 1). Extracolonic findings were consistent with an ongoing postoperative ileus, with small-volume ascites and no pneumoperitoneum. The sigmoid thickening was attributed to an inflamed, healing anastomosis. Total parenteral nutrition (TPN) was commenced, and a nasogastric tube placed on free drainage to allow resolution of the ileus and anastomotic inflammation before resuming enteral nutrition.

On postoperative Day 10, the patient developed a fever of 38.1°C, with all other vital signs remaining within normal limits. Auscultation of the right lung base revealed new crepitations. The abdomen was distended but soft, with no signs of peritonism. There were no other localizing features of infection and no clinical evidence of venous thromboembolism. Blood tests demonstrated an elevated white cell count (15.3 × 10^9^/L) with predominant neutrophilia (14.3 × 10^9^/L), while C-reactive protein remained elevated at 136 mg/L, trending down from 201 mg/L the previous day. Arterial blood gas analysis showed a pH of 7.49 with normal bicarbonate and lactate levels.

Following the above examination findings and results intravenous fluids were administered, intravenous (IV) antibiotics were started as per hospital antibiotic guidelines, and chest X-ray was requested for presumed hospital acquired pneumonia.

**Figure 2:**
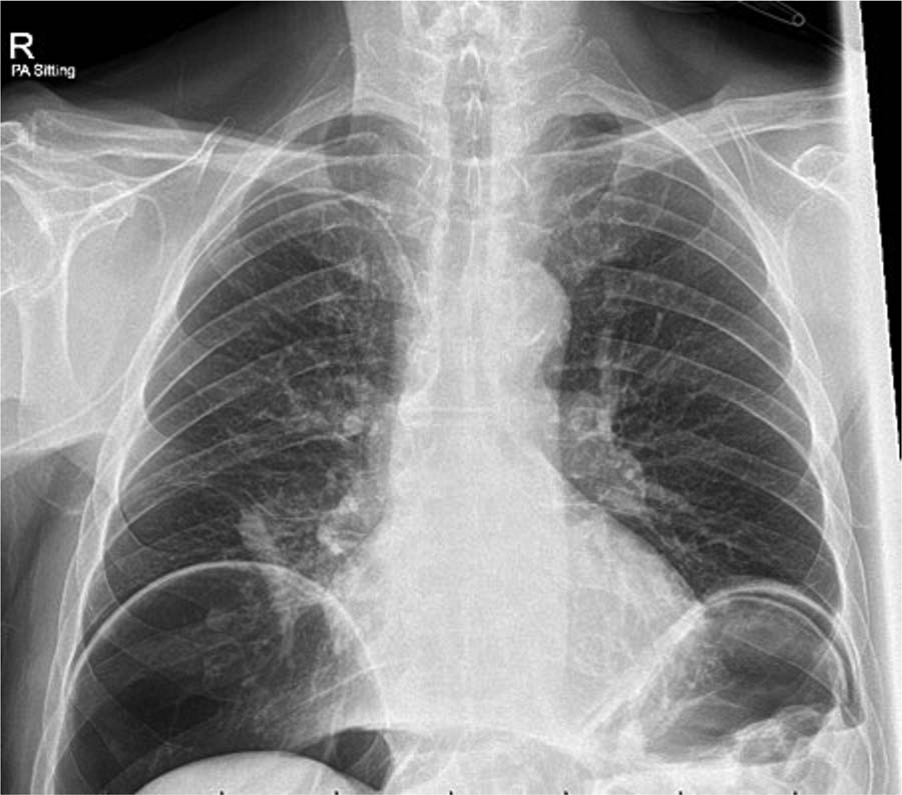
Day 10 chest x-ray demonstrating massive pneumoperitoneum.

**Figure 3:**
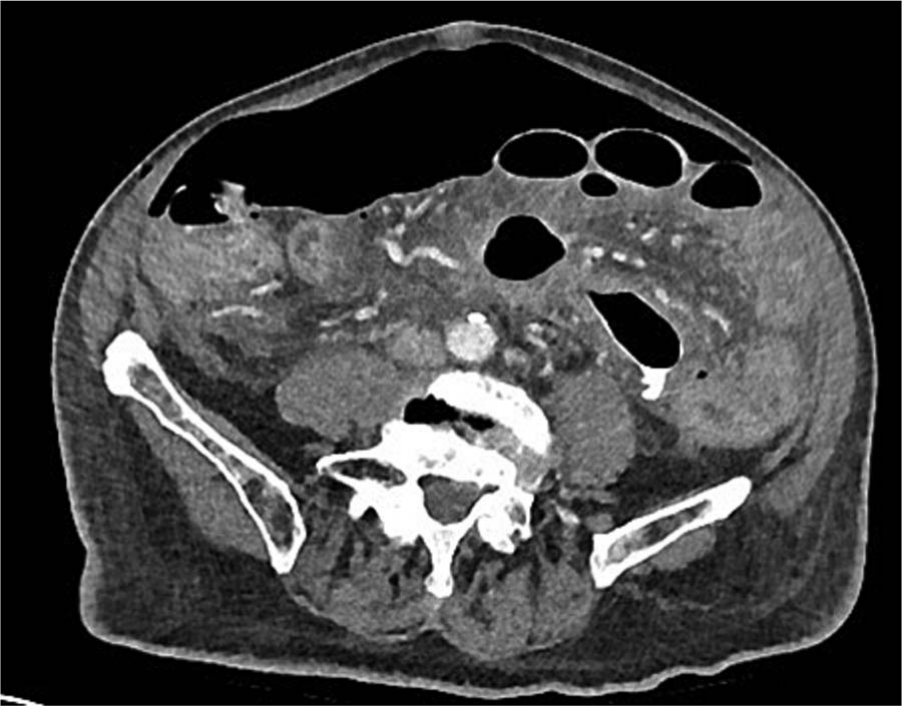
Day 10 CT scan demonstrates ileus with large volume pneumoperitoneum with free air around anastomosis.

A chest x-ray confirmed new right lower lobe consolidation and demonstrated interval development of massive pneumoperitoneum compared to the CT scan 3 days prior (Fig. 2). A repeat CT scan was therefore undertaken, revealing large volume pneumoperitoneum with free gas locules but no rectal contrast extravasation at the region of the anastomosis (Fig. 3).

After discussion of operative management, the patient alongside consultation with family elected against further surgical intervention. Therefore he was treated with IV antibiotics and continued on TPN for nutrition.

Over the following 2 weeks, the patient’s vital signs remained in normal range and abdomen was benign on serial examination. Clinical improvement was evidenced by the reduction in nasogastric output and return of bowel movements. Enteral feeding was established and TPN ceased. IV antibiotics were also stopped following a 7 day course. Repeat chest X-ray at day seventeen of admission demonstrated a reduction in volume of pneumoperitoneum. He was successfully discharged home 24 days following the initial sigmoid colectomy.

## Discussion

Although the term ‘anastomotic fart’ has been informally used by surgeons for decades, it is absent from the formal medical literature. Its inclusion in this report may help standardize terminology for this diagnostically challenging, yet often benign, phenomenon.

This case illustrates the concept of an ‘anastomotic fart,’ defined as the passage of gas through an anastomosis into the peritoneal cavity without passage of significant faecal contamination. The absence of gross contamination and subsequent clinical improvement suggests a transient gas leak rather than a full anastomotic disruption. Sequential cross sectional imaging established a clear timeline of the development of new pneumoperitoneum between post-operative Day 7 and 10.

Despite the large-volume of pneumoperitoneum, this case demonstrates that conservative management was appropriate. Recognition of this phenomenon is essential to avoid unnecessary re-laparotomy in clinically stable patients.

Consensus on the most appropriate management of anastomotic leak remains variable, largely due to varying definitions and terminology [1]. The presence or absence of a proximal diverting stoma also influences management decisions. Traditionally, leaks have been managed operatively or interventionally, including re-exploration with resection of the anastomosis, re-fashioning, formation of diverting or end stoma or drainage of contamination [1]. More recent studies have evaluated the role of conservative management in selected patients [3, 4]. We propose that conservative management may be appropriate for clinically stable patients, with leakage predominantly of gas, even in the setting of significant pneumoperitoneum. Nonetheless, meticulous serial assessment and multidisciplinary input remain vital, as anastomotic leaks with contamination still demand urgent intervention [1–3].
